# Bibliometric Analysis of Health Technology Research: 1990~2020

**DOI:** 10.3390/ijerph19159044

**Published:** 2022-07-25

**Authors:** Xiaomei Luo, Yuduo Wu, Lina Niu, Lucheng Huang

**Affiliations:** College of Economics and Management, Beijing University of Technology, Beijing 100124, China; xmluochina@bjut.edu.cn (X.L.); wydq@emails.bjut.edu.cn (Y.W.); niulina@emails.bjut.edu.cn (L.N.)

**Keywords:** healthy technology, bibliometrics, Citespace, VOSviewer, emerging research topic, research frontier

## Abstract

This paper aims to summarize the publishing trends, current status, research topics, and frontier evolution trends of health technology between 1990 and 2020 through various bibliometric analysis methods. In total, 6663 articles retrieved from the Web of Science core database were analyzed by Vosviewer and CiteSpace software. This paper found that: (1) The number of publications in the field of health technology increased exponentially; (2) there is no stable core group of authors in this research field, and the influence of the publishing institutions and journals in China is insufficient compared with those in Europe and the United States; (3) there are 21 core research topics in the field of health technology research, and these research topics can be divided into four classes: hot spots, potential hot spots, margin topics, and mature topics. C21 (COVID-19 prevention) and C10 (digital health technology) are currently two emerging research topics. (4) The number of research frontiers has increased in the past five years (2016–2020), and the research directions have become more diverse; rehabilitation, pregnancy, e-health, m-health, machine learning, and patient engagement are the six latest research frontiers.

## 1. Introduction

Health technology refers to drugs, equipment, operations, procedures, and organizational and support systems that prevent, diagnose, or treat disease, as well as promote health and provide rehabilitation or medical care [1,2,3,4,5]. Health technology, as an emerging concept, has been put forward and widely used in the last thirty years. Due to the explosive growth of the publication numbers in the field of health technology (as shown in Section 3.1), it is particularly important to review these publications.

The terminology used to define technological changes in healthcare and medicine has changed from medicine technology (1940s–1970s), to healthcare technology (1980s), to health technology (1990s–present) [6,7]. In this era of technology revolution, new health technologies continue to emerge. Virtual reality, wearable health detection, implantable sensors, and 3D printing are widely applied in the health industry [8,9,10,11]. The application of these technologies can promote great changes and thoroughly improve the capability of the whole healthcare system [12,13,14,15,16,17,18].

Some scholars adopted qualitative methods to review some health technology research: [19,20]; others applied bibliometric methods to review some health technology systems and the application of emerging technologies in the health sector [21,22,23,24,25,26,27,28]. However, these studies only focus on a specific piece of technology or application. The lack of a comprehensive picture of the current status in the field of health technology research makes researchers’ overall understanding limited.

Considering the above research gaps, this paper aims to conduct a comprehensive bibliometric analysis of health technology literature, and determined the following main objectives: (1) Analyze the publishing trend of papers in the field of health technology. (2) Assess and visualize the current status of health technology research and cooperation from three aspects: authors, journals, and institutions. (3) Use various bibliometric methods to identify the core research topics, emerging research topics, and frontier evolution trends of health technology research. Overall, this paper provides panoramic knowledge support for researchers in this field.

## 2. Materials and Methods

### 2.1. Methodology and Tools

Bibliometrics refers to the method of applying mathematical statistics to quantitatively analyze the temporal and spatial distribution properties of scientific documents in a specific field, which can realize the scientific transformation of documents from data to knowledge [29,30]. In bibliometrics, there are two main procedures: performance analysis and science mapping [31]. Performance analysis is based on bibliographic data to assess the impact of groups of scientific actors (countries, institutions, and researchers) and their activities. Science mapping aims to show the knowledge structure, dynamic evolution, and trends in the research field, which can provide spatial representation through physical proximity and relative position to show the relationships between disciplines, fields, papers, or authors [32]. The most commonly used analytical methods of bibliometrics are documents co-citation and co-word analysis. Documents co-citation [33] refers to mapping the knowledge structure of the research field through the commonly cited paired documents. Co-word analysis [34] is a kind of content-analysis technology, which directly deals with the term set shared by documents and maps relevant documents through the interaction of key terms. The results of co-citation or co-word analysis can be used for a variety of purposes, such as identifying current research hotspots and frontiers and analyzing the evolution and trend of knowledge structure [35,36,37].

The VOSviewer [38] software developed by Leiden University in the Netherlands can realize the mining of literature authors, journals, countries, and other information through bibliometrics, and can also carry out visual analysis by constructing citation networks and co-occurrence networks. This software has advantages in the accuracy of information mining, network density, and cluster visualization. In this paper, VOSviewer software was used to mine the information of authors, journals, and institutions of health technology literature, as well as to analyze the map of the core author group, key points of published journals, and the cooperative network of institutions.

Citespace [39] software developed by Professor Chen Chaomei has been widely used to identify research topics and research frontiers, and has become a popular tool in bibliometrics research. In this paper, the “TOP N” algorithm of Citespace software was used to extract the top 30 high-frequency keywords in each time slice, and the co-occurrence map and co-occurrence matrix of high-frequency health technology keywords from 1990 to 2020 were generated. Then, Callon’s clustering rules [40] and a strategic coordinate graph analysis method [41] were combined to identify the core research topics and emerging research topics of health technology. Then, the Burst Detection function of Citespace was used to extract the burst keywords to identify the research frontiers in the field of health technology.

In the process of identifying emerging research topics, it is necessary to establish strategic coordinates by taking the attention index of research topics as the horizontal axis and the novelty index as the vertical axis. According to the novelty and attention of each research topic, as well as its distribution in the four quadrants, the emerging research topics could be identified. The calculation formulas of novelty and attention are shown as Formulas (1) and (2):

(1) $${\mathrm{ND}}_{i}=\frac{1}{M}\sum_{j=1}^{m}Y_{\mathrm{ij}}-\frac{1}{N}\sum_{g=1}^{n}Y_{g}\;(\;i=1,\;2,\;\ldots \ldots ,\;K)$$

Formula (1): ${\mathrm{ND}}_{i}$ represents the novelty degree of the i cluster; $\frac{1}{M}\sum_{j=1}^{m}Y_{\mathrm{ij}}$ represents the average annual co-occurrence of M keywords in the i cluster; $\frac{1}{N}\overset{n}{\underset{g=1}{\sum }}Y_{g}$ represents the annual average of N keywords co-occurrence.

(2) $$C_{i}=\frac{1}{M}\sum_{j=1}^{m}F_{\mathrm{ij}}-\frac{1}{N}\sum_{g=1}^{n}F_{g}\;(\;i=1,\;2,\;\ldots \ldots ,\;K)$$

Formula (2): $C_{i}$ represents the attention of the i cluster; $\frac{1}{M}\sum_{j=1}^{m}F_{\mathrm{ij}}$ represents the average co-occurrence frequency of M keywords in the i cluster; $\frac{1}{N}\overset{n}{\underset{g=1}{\sum }}F_{g}$ represents the average frequency of co-occurrence of N keywords.

### 2.2. Data Collection

Health technology is aimed at the whole population, including healthy people, sick people, and sub-healthy people. Based on this, the paper creates the retrieval strategy of health technology as TI = (healthy (#1) AND technology (#2) AND the whole population (#3)); the details are listed in Table 1. This paper was retrieved in the Web of Science (WOS) database with the above search expression on 1 March 2021. We limited the database to SCI-E and SSCI, the literature type to “‘Article’ and ‘Review’”, and the language to “English”. The timespan was set as “1 January 1990–31 December 2020”. The retrieval results showed that a total of 8418 pieces of literature met the retrieval criteria.

**Figure 1:**
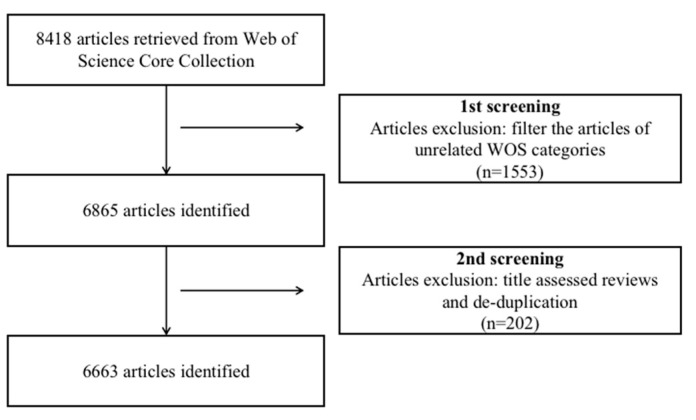
A flowchart representing retrieval strategies for health technology articles from the WOS database and the inclusion criteria for the study.

To ensure the reliability of the data, we screened the retrieved articles in two stages by referring to the Preferred Reporting Items for Systematic Reviews and Meta-Analyses (PRISMA) [42] and the screening process of Qi [43] and Selva-Pareja [44]. In the first stage, the filter provided by the WOS database was used to get rid of documents in unrelated categories, including veterinary sciences, food science technology, zoology, construction building technology, and plant science. The second stage was performed with the article’s titles to de-duplicate and clean the remaining documents [44]. After the two-stage screening (figure), we deleted the documents that were irrelevant to health technology, and the technical application object, which does not belong to the whole population category, and 6663 documents on health technology were obtained. The specific process can be seen in the flow chart (Figure 1).

## 3. Results

### 3.1. Publication Trend Analysis

**Figure 2:**
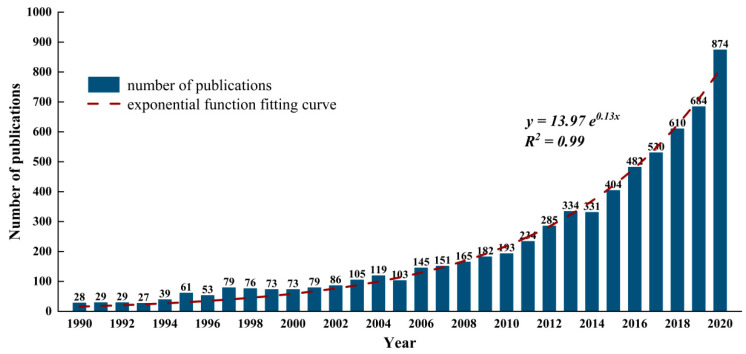
Number of publications and growth trend.

This paper uses bibliometrics to count the number of publications each year and uses an exponential function to fit (Figure 2) [45]. From 1990 to 2020, the number of publications in the field of health technology increased exponentially.

### 3.2. Distribution Analysis of Authors, Journals, and Institutions

The core author group is a collection of authors who have published considerable amounts of literature and have great influence. Analyzing the core author groups can reveal the contribution degree of major researchers and their teams in the research field. Using VOSviewer to analyze the authors, the top 10 authors in the field of health technology by volume were obtained (Table 2). As can be seen from Table 2, Marie-Pierre Gagnon is the author with the most literature in the field of health technology, with a total of 9 publications. Therefore, according to the formula of Price’s Law [46] $M=0.749\sqrt{N_{\max}}$, authors who have published three or more articles (M = 2.247) are considered core authors in the field of health technology. According to literature statistics, there are 323 core authors in the health technology research field, and the total number of published articles is 1080, accounting for 16.2% of the total number of published articles (6663). This is far less than 50%, indicating that there are many authors in the field of health technology, but the stable core author group has not been formed in this research field.

The VOSviewer software was used to analyze the health technology publishing journals, and the results showed that there were 2315 health-technology-related journals in total. Table 3 shows the top 10 journals, publishing countries, number of published articles, and citations, suggesting that: (1) From the country of publication, among the top 10 journals in terms of cumulative number of published articles, there are 5 journals from the United Kingdom, 2 journals from Canada, and 1 journal from the United States, Switzerland, and the Netherlands, respectively, but no journals from China, suggesting that Chinese journals of health technology have fewer publications and less international influence. (2) *PLOS ONE*, *BMC HEALTH SERVICES RESEARCH*, and *BMC PUBLIC HEALTH* are the three journals with the highest number of publications and citations. In terms of the amount of literature, there is a certain gap between the number of publications of adjacent journals, but the gap is small. (3) *SOCIAL SCIENCE & MEDICINE*, *QUALITY OF LIFE RESEARCH*, and *INTERNATIONAL JOURNAL OF TECHNOLOGY ASSESSMENT IN HEALTH CARE* are the three journals with the highest number of citations per article.

The VOSviewer software was used to analyze the publishing institutions, and the results show that there are 8124 research institutions in the field of health technology. The top 10 research institutions in terms of publishing volume are shown in Table 4. As can be seen from Table 4, Harvard University, the University of Toronto, and Johns Hopkins University are the top three research institutions with the most literature. From the country of origin of research institutions, 4 out of the top 5 research institutions, 8 out of the top 10 research institutions, and all the top 5 research institutions in terms of literature quantity are all from the United States. There is no institution from China among the top 10 health technology research institutions, suggesting that the literature publishing and international influence of Chinese health technology research institutions are weak.

**Figure 3:**
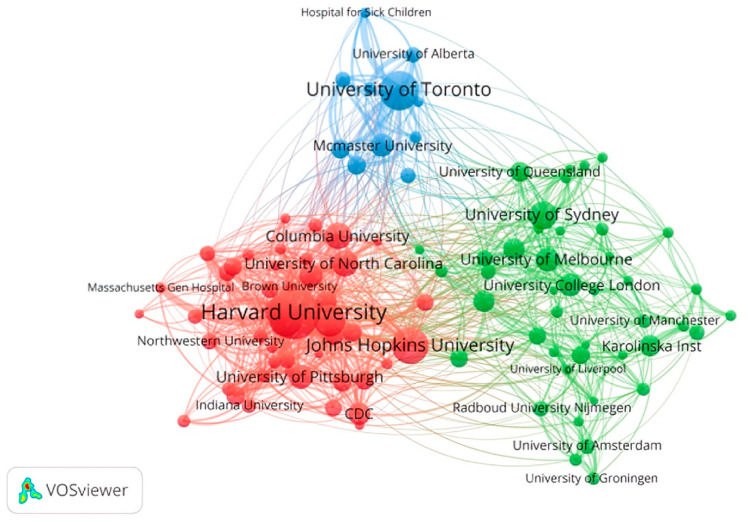
Institutional co-authorship map of health technology research. The node area represents the number of articles issued by institutions, the links between nodes represent the cooperation between institutions, and the distance and the width of the links between nodes represent the cooperation intensity between institutions.

The network spectrum of institutional cooperation can reflect the academic influence and inter-institutional cooperation of research institutions in this field [22]. Therefore, this paper carries out a network atlas analysis of the cooperation among the top 100 research institutions in terms of the number of publications (the lowest number is 22) (Figure 3). From Figure 3, inter-agency cooperation in the field of health technology has formed three obvious “clusters”, namely a red node cluster with American research institutions as the core, a green node cluster with European and Australian research institutions as the core, and a blue node cluster with Canadian research institutions as the core. Among them, the red node cluster is centered on Harvard University, with intensive internal connections, indicating that the cooperative network of health technology research institutions in the United States is developed. The number of institutions in the blue node cluster is small, indicating that Canadian health technology research institutions have relatively little academic influence, but they still form a research cluster centered on the University of Toronto. There is no large node in the green node cluster, and the internal connections are relatively scattered, indicating that European and Australian health technology research institutions have fewer publications and academic influence, and the inter-institutional cooperation network is underdeveloped.

## 4. Research Topics and Evolution Analysis

### 4.1. The Research Topics Analysis

**Figure 4:**
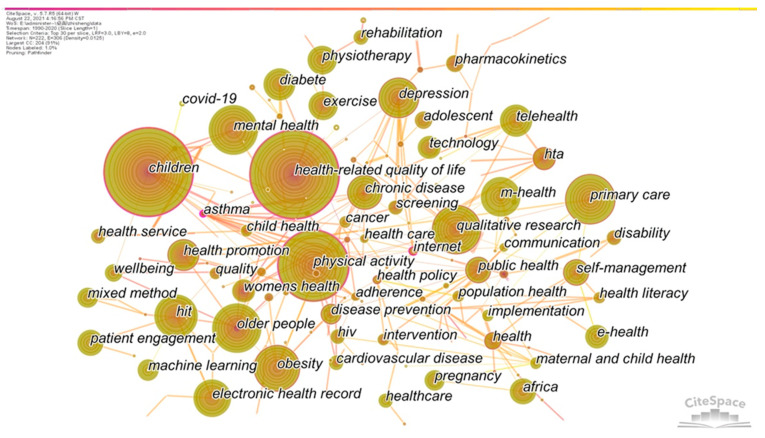
Keywords co-occurrence map of health technology research.

The literature data were imported into Citespace. “Time Slicing” was set as “1990–2020”, one year was taken as a Time slice, and the “TOP N” algorithm was used to extract the top 30 high-frequency keywords in each slice. The co-occurrence map of high-frequency keywords of health technology from 1990 to 2020 (Figure 4) and the co-occurrence matrix were generated. Then, referring to the clustering rules of Callon [40], the 222 generated high-frequency keywords were divided into 42 clusters according to the co-occurrence relationship between keywords.

According to the keywords contained in the clusters, we summed up the research topics on behalf of the clusters, excluded irrelevant and ambiguous clusters, and finally, we formed 21 core research topics of the health technology research field (Table 5). These core research topics represent the main research interests of researchers and groups in the health technology research field.

**Figure 5:**
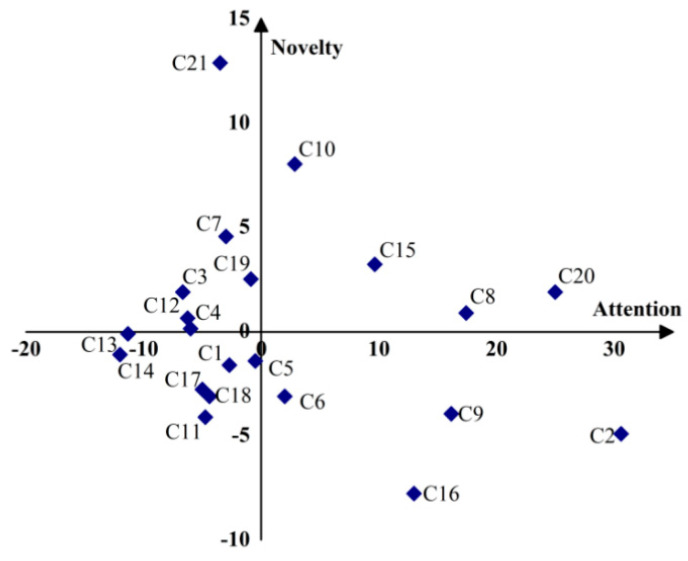
Strategic diagram of health technology research topics.

Based on the core research topics we have identified, the strategic diagram method proposed by Law et al. [41] was used to establish the research topics’ strategic diagram of attention and novelty degree. According to the index meaning and calculation formula of novelty degree and attention, the strategic coordination of research topics of health technology was obtained (Figure 5). According to the degree of novelty and attention of each topic and its distribution in four quadrants of the strategic diagram, we divided the 21 core research topics into four categories: hot spots (the first quadrant), potential hot spots (the second quadrant), marginal topics (the third quadrant), and mature topics (the fourth quadrant). It can be seen from Figure 5 that: (1) telemedicine (C8), digital health technology (C10), electronic health record (C15), and health information technology (C20) are located in the first quadrant, with the characteristics of high attention and high novelty, falling into the category of the current research hot spots in the field of health technology. Among them, digital health technology is the most novel research hot spot, and telemedicine and health information technology are the two research hot spots receiving the most attention. (2) The six topics, including assistive technology (C3), adolescent health (C7), public health (C19), and COVID-19 prevention (C21), are located in the second quadrant, with the characteristics of low attention and high novelty. These research topics are relatively novel and have the potential to become hot spots as they are paid more attention. Among them, the three research topics of adolescent health, public health, and novel coronavirus prevention and control with relatively high attention are most likely to become new hotspots in the future. (3) Seven topics, including health management (C1), disease prevention (C5), mass spectrometry (C13), and female health (C17), are located in the third quadrant. They have the characteristics of low attention and low novelty. They are marginal research topics that have appeared earlier, but receive less attention. Among them, the novelty and attention of health management and disease prevention are relatively good, and they could be expected to develop into mature topics as they are paid more attention in the future. (4) The four topics of child health (C2), risk assessment (C6), health technology assessment (C9), and physical therapy (C16) are located in the fourth quadrant. They have the characteristics of high attention and low novelty, indicating that these research topics appeared earlier and have developed into mature research topics. Among them, child health, health technology assessment, and physical therapy still maintain a high degree of attention, and may continue to focus on research in combination with other hot spots in the future.

Among the 21 core research topics, the novelty degree of C21 (COVID-19 prevention) and C10 (digital health technology) is significantly higher than that of other research topics. Therefore, we identified these two research topics as emerging research topics in the field of health technology. Next, we will elaborate on these two emerging research topics based on the latest literature and keywords of the topics.

C21 (COVID-19 prevention): The sudden outbreak of COVID-19 drew the attention of all countries to global health and epidemic prevention systems, and accelerated the digital transformation of public health governance and healthcare to a certain extent. Digital technologies such as big data, artificial intelligence, and cloud computing have played an important role in COVID-19, while digital health services such as telemedicine have effectively prevented cross-infection. Scott identified digital health technologies that can be implemented in the response and prevention of COVID-19, based on a review of relevant research literature and technology news, including telemedicine and mobile nursing (for COVID-19 and routine nursing), tiered telemonitoring, tele-critical care, robotics, and artificial intelligence for monitoring [47]. Aminullah studied the impact of policy innovation on the scale and speed of COVID-19 transmission by building a system dynamics model, and also studied the impact of the emergence of innovative health technologies, including medical devices, devices, drug development, and vaccines, on the health system in their research [48]. Magrabi reviewed existing health technology assessment methods and evaluated three digital health technologies for COVID-19 response, namely mature technologies, new technologies deployed on a large scale, and new technologies deployed on a small scale, based on their technology maturity and scale of implementation [49]. COVID-19 is a disaster, but it also presents an opportunity to adapt and use the HTA process creatively and constructively, as a tool for transforming entire health systems and creating value for society in the post-COVID-19 era [50].

C10 (Digital Health Technology): The range of digital health technologies includes m-health, health information technology (HIT), wearable devices, telemedicine, personalized medicine, etc. WHO believes that the use and promotion of digital health technologies can help people achieve higher health standards and access healthcare services, and has been promoting and calling for the use of digital health technologies to promote the improvement of people’s health and health systems worldwide. In 2019, WHO released the Global Strategy for Digital Health (2020–2024), which identified the priority of digital health strategies and affirmed the potential of digital health technologies to support the development of the healthcare sector in all countries. Digital health technology has been widely used in the past decade. Mahajan assessed the use of digital health technologies among US adults in two areas, namely online search for health information and access to healthcare services, using data from the National Health Interview Survey from 2011 to 2018, and projected future usage [51]. Digital health technology not only offers assistance for disease prevention, early disease diagnosis, and management of chronic disease (medical service suppliers: doctors, medical institutions, etc.), but also improves efficiency and quality, reduces the cost of healthcare services, and provides the patient with personalized health care (medical service demand: patients and healthy people, etc.). Using the balanced employment and life (MABEL) questionnaire data of 7043 doctors, Zaresani studied the impact of doctors’ use of digital health technology on improving their job satisfaction and promoting work–life balance [52]. The application of the Internet to elder care and the use of the Internet by older persons is an important means of promoting digital health technologies. Sun studied the current situation and influencing factors of the elderly’s Internet use and assessed the elderly’s demand for digital health technologies [53]. Digital health technology needs to be driven by health data to understand health behaviors, or to apply the data to the diagnosis and treatment of diseases [54]. Therefore, in the past few years, digital medical services have increased exponentially, and are not regulated, which has aroused people’s concerns about data security and privacy [55]. While using digital health technology to provide healthcare services, it is also necessary to ensure the security of personal data, so as not to face risks due to the disclosure of sensitive health information.

### 4.2. Research Frontiers Analysis

The research frontier represents the most prospective and potential research direction in scientific research. To promote health technology innovation, it is necessary to implement and apply frontier technologies. The theoretical and technological breakthroughs of frontier disciplines can provide power and support for the development of health causes and form new economic growth points. The Burst Detection function of CiteSpace can detect burst keywords with an explosive increase in citations in a specific period, and the detection results can be used as the basis for analyzing research frontiers [56].

**Figure 6:**
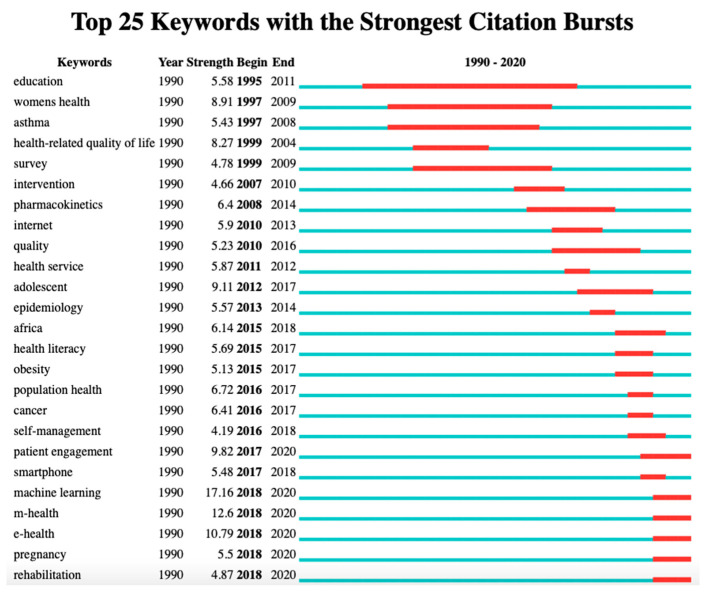
Health technology keywords with the strongest citation bursts.

This paper uses the “Burst Detection” function of Citespace to extract the emerging keywords in the field of health technology. The results are shown in Figure 6. Keywords that emerged over a recent five-year period (2016–2020) and continued to emerge until 2020 were identified as latest health technology research frontiers, as shown in Figure 6. (1) There are six latest research frontiers in the field of health technology: rehabilitation, pregnancy, e-health, m-health, machine learning, patient engagement, and the time to which a burst continues nowadays. (2) Considering Burst Strength, the top three keywords were machine learning (Burst Strength 17.16), mobile health (Burst Strength 12.6), and e-health (Burst Strength 10.79), indicating that the above research frontiers are highly concerned in the field of health technology, and these three research frontiers are the most influential in the field of health technology research; (3) From the perspective of the time period, 15 burst keywords were extracted before 2015 (period 1: 1990–2015), with an average annual number of one, while 10 burst keywords were proposed in the past five years (period2: 2016–2020), with an average annual number of two. The number of burst keywords per year in period 2 is twice that of period 1, indicating that the number of research frontiers has increased in the past five years, and the research directions have become more diverse.

### 4.3. Industry 4.0 Technologies Supporting the Health Sector

Based on the results of Section 4.1 and Section 4.2, we have noticed that some technical keywords closely related to industry 4.0 have emerged as core/emerging research topics or research frontiers and occupy an important position in the knowledge structure in the health technology research field, such as telemedicine, digital health technology, electronic health record, health information technology, smart phones, e-health, m-health, and machine learning. We think these keywords deserve widespread attention, and considering the practical significance and research value of industry 4.0 technology applied in health sector, we will describe industry 4.0 and industry 4.0 technologies supporting the health sector in this section, in order to provide a reference for future researchers.

Industry 4.0, first proposed in 2011 [57], refers to the intelligent production process in the manufacturing industry, which mainly covers the Internet of things (IoT), cloud computing, big data, artificial intelligence, etc. Industry 4.0 is characterized by the extensive use of intelligent objects in highly reconfigurable and fully connected industrial product service systems [58], which has brought unprecedented damage to all traditional production/service systems and business models (value chains) and accelerated the demand for activity redesign and digitization [59,60,61]. With the expansion of these emerging or disruptive technologies, the disruptive and transformative wave of industry 4.0 has incredibly transformed many industries such as education, energy, agriculture, and healthcare. Due to the impact on the healthcare industry, a new concept called “healthcare 4.0” has been formed [62,63]. The Internet of things, block-chain, cloud computing, artificial intelligence, and other industry 4.0 technologies have brought amazing progress to healthcare industry. The role of industry 4.0 in healthcare is extraordinary because it reduces the associated time and cost [12], provides more effective and efficient healthcare services, and leads to the implementation of better solutions [13], including the high security and privacy of electronic health records of patient data, allowing doctors or healthcare personnel to conduct remote and real-time access and diagnosis [64,65]. Therefore, although healthcare 4.0 is considered highly complex and expensive, many developed countries have begun to accept it [66,67]. To sum up, considering the importance and future research value of industrial 4.0 technologies such as Internet of things, block-chain, cloud computing, and artificial intelligence for the health industry, we will overview the main applications and latest research of these technologies in the health sector, as follows:

**The IoT and big data.** The IoT technologies applied in the healthcare industry mainly include three categories: Wearable IoT, fabric and flexible sensors, and ambient IoT [64]. With the rapid development of the Internet of things, wearable devices and sensors continue to generate a large amount of data about our physical and mental health—“big data”—which can provide new insights and accurate medical solutions, as well as support for telemedicine [68,69]. These sensors, the Internet of things, and big data are redesigning the technical, economic, and social expectations of modern medical services [70]. Kwon reviewed the latest technological progress of wearable sensors and integrated portable electronic devices for sleep monitoring applications. Based on the current challenges faced by wearable sleep monitoring technology, they put forward prospects for the development of new technologies [71]. Dinh-Le reviewed wearable health technology in a wide range, overviewed the innovation in the wearable technology field in the current Electronic Health Record (EHR), and discussed the key challenges and emerging solutions faced by this rapidly developing field [72]. However, the data collected by m-health technologies such as wearable devices and smart phone health applications are faced with the problem of privacy protection. Suver discussed the consequences of digital data collection and the trade-off between privacy and public health benefits [73].

**Block-chain.** Block-chain plays a vital role in modern healthcare systems, and is considered to be one of the most transversal and promising technologies [74,75,76]. The data source, robustness, decentralized management, security, and privacy of the block-chain and the immutability of stored data are the main reasons why the block-chain attracts the attention of the healthcare industry [74,77]. Due to its technical characteristics, the application of block-chain in healthcare industry has provided some benefits, but there are also implementation and supervision problems. Therefore, the practicality of block-chain technology in the medical industry has been questioned. The current limitations are mainly related to model performance, implementation constraints, and costs [75,78]. Many industries have adopted or are adopting necessary technologies to meet users’ demand for instant information, but the healthcare sector has fallen behind in this regard and is still facing the challenge of establishing a high-performance, scalable, and patient-centered information storage and exchange environment [74]. Block-chain has been widely considered by scholars in the health technology research field. Zhuang built a general block-chain architecture that can provide data request, authorization, data exchange, and tracking functions for the development of healthcare applications, and proved the feasibility of the architecture through experiments (performance tests) [79]. Ichikawa developed an m-Health system for treating cognitive behavioral therapy for insignia (CBTI) based on the block-chain storage platform and evaluated the anti-tampering ability of the data collected [80]. Abunadi put forward the block-chain and business process management (BBPM) system in healthcare. The system has the advantages of both block-chain and business process management systems, which can effectively help alleviate the spread of COVID-19. However, the BBPM system still faces many limitations and needs to be improved in the future to become more novel, energy saving, and scalable [81].

**Artificial Intelligence.** Artificial intelligence has a significant impact on medical care. The application of artificial intelligence in the health sector is expected to change the method of diagnoses, prevention and treatment, interaction with technology [82], including automatically handling routine tasks [83], accurately identify patient needs [84], and assist clinicians in decision-making [85]. During the COVID-19, artificial intelligence technology also played a crucial role in assisting patients’ treatment, optimizing clinical trials of drugs and vaccines, and managing the supply chain of medical departments [86,87,88]. Based on the systematic review of the literature related to Artificial Intelligence Health Technology (AIHT), Bélisle-Piponetal put forward that AIHT has particularity in five aspects: nature, scope, increased expectations, new ethical challenges, and new evaluation constraints, which make artificial intelligence stand out in HTA [89]. Hendrix used a broad value framework to evaluate the economic value of the potential utilization of artificial intelligence and discussed how AI challenges the traditional health technology assessment (HTA) method and the future research direction of clinical AI economic value assessment [90]. Rowe believed that artificial intelligence could be used to improve the health and well-being of adolescents, and introduced how some emerging AI technologies could provide personalized health intervention support for adolescents [91].

**Cloud Computing.** With the help of cloud computing, healthcare organizations and hospitals can ensure the storage of massive data and computing power, and access to all kinds of information of patients, doctors, different hospitals, and health organizations anytime and anywhere. Moreover, cloud storage data do not need upfront costs, but only need to pay for user resources, thus saving a great deal of money [92]. Cloud computing is also widely used in technology integration to support the development of new algorithms or technologies. Yu proposed a new algorithm of human identification for healthcare applications using mobile edge computing (MEC), and the algorithm realizes efficient patient identification by running cloud servers at the edge of mobile networks [93]. Lakshmi proposed a method to realize remote patient monitoring through cloud-based IoT medical sensors, collect biomedical data through wearable sensors, and directly transmit patient data to cloud intensive care systems to remotely monitor health status [94]. However, remote health monitoring faces the challenge of privacy protection. The cloud-based privacy protection system proposed by Alabdulatif enables all analysis and calculation to be carried out on encrypted data, and the framework operates in a completely independent way, which can be used as a reference to solve privacy protection obstacles [95].

## 5. Conclusions

In the 1990s, health technology became an independent term. In the following three decades, the amount of literature in the field of health technology increased exponentially, but no research could provide a comprehensive picture including the literature distribution, research topics, and frontiers, nor promote the further development of this research field. Based on this, this paper used bibliometrics, cluster analysis, and strategic coordinate analysis to analyze the research trend, causes, and distribution. It identified and discussed the research topics (core topics and emerging topics) and frontiers of this research field, and obtained the following conclusions:

(1) Since the 1990s, health technology has been widely used as an independent term. The health technology literature quantity has shown an exponential rapid growth trend from 1990 to 2020, and the research field has developed rapidly.

(2) There are many authors in the field of health technology worldwide, but this research field has not formed stable core authors yet. Cooperation between institutions has formed three distinct “clusters”. Compared with Europe and the United States, China’s international influence in the health technology research field is relatively weaker and is at disadvantage in publishing institutions and journals.

(3) Through cluster analysis of co-occurrence keywords and strategic diagram analysis, we obtained 21 core research topics of health technology, and divided these research topics into four classes (hot spots, potential hot spots, marginal topics, and mature topics). The novel degree of C21 (COVID-19 prevention) and C10 (digital health technology) is significantly higher than other research topics, which are identified as emerging research topics of health technology and were elaborated on with the latest studies.

(4) The number of research frontiers of health technology increased in a recent five-year period (2016–2020), suggesting the research directions are more diversified. Among them, machine learning, m-health technology, and e-health technology are the three frontiers with high Burst Strength and attention. In addition, many keywords we found, as components of the research topics and frontiers, are related to industry 4.0 technologies, so we provided an overview of the main applications and recent research of these important Industry 4.0 technologies used in the health sector.

This study may contribute to existing research in three aspects. Firstly, this study is the first bibliometric analysis of health technology using scientific mapping tool software (CiteSpace and VOSviewer). We used bibliometric analysis to provide a new insight that was not conducted comprehensively in previous studies. Secondly, through elaborating the analysis results of clustering, strategic coordinate analysis, and burst detection we performed, researchers of health technology could better understand the core research topics, emerging research topics, and research frontiers of this field. Thirdly, the analysis framework and conclusions of this study will provide the research basis and direction for bibliometric analysis of related research. Overall, this study is a comprehensive review of health technology literature; it can also serve as a solid foundation for future research.

## Figures and Tables

**Table 1:** Health technology publications retrieval strategy.

Retrieve	Retrieval Expression
#1	TI = (“health *” or “well *” or “physi *” or “sound*” or “fit *” or “wholesome *”)
#2	TI = (“technology *” or “technique” or “facility” or “device *” or “apparatus” or “tool *” or “equipment” or “machine *” or “means” or “approach *” or “method *” or “solution *” or “procedure *” or “way *”)
#3	TI = (“man *” or “wom?n” or “person” or “people” or “child *” or “adult” or “teenager” or “elder” or “human *” or “citizen” or “population” or “sufferer” or “patient *” or “invalid” or “disease *” or “ill *” or “pathema” or “ailment *” or “malady” or “sick *” or “weak *” or “non-health” or “unhealth *” or “unwell *”or “unsound *” or “indisposed” or “uncomfortable *” or “discomfort *” or “sub-health” or “semi-health”)

**Table 2:** Top 10 authors in the field of health technology.

No.	Authors	Number of Publications	Citations
1	Marie-Pierre Gagnon	9	168
2	France Legare	8	265
3	Brian Maccrindle	7	245
4	Ding Li	7	11
5	VR Young	6	261
6	Francois-Pierre Gauvin	6	229
7	Trudy Van Der Weijden	6	157
8	Marie Desmartis	6	156
9	Johanne Gagnon	6	156
10	Julia Abelson	6	139

If the number of studies is the same, we ranked based on the citations, the same as below.

**Table 3:** Top 10 academic journals in the field of health technology research.

No.	Journal	Country	Number of Publications	Citations
1	*PLOS ONE*	The United States	125	1405
2	*BMC HEALTH SERVICES RESEARCH*	The United Kingdom	111	1196
3	*BMC PUBLIC HEALTH*	The United Kingdom	93	1190
4	*BMJ OPEN*	The United Kingdom	87	503
5	*JOURNAL OF MEDICAL INTERNET RESEARCH*	Canada	66	948
6	*INTERNATIONAL JOURNAL OF ENVIRONMENTAL* *RESEARCH AND PUBLIC HEALTH*	Switzerland	53	331
7	*JMIR MHEALTH AND UHEALTH*	Canada	48	320
8	*INTERNATIONAL JOURNAL OF TECHNOLOGY* *ASSESSMENT IN HEALTH CARE*	The United Kingdom	45	919
9	*QUALITY OF LIFE RESEARCH*	Netherlands	40	1042
10	*SOCIAL SCIENCE & MEDICINE*	The United Kingdom	37	991

**Table 4:** Top 10 research institutions of health technology research.

Institution	Cluster	Number of Links	Linking Strength	Number of Publications	Citations
Harvard University	1	60	102	145	3855
University of Toronto	3	35	76	123	2239
Johns Hopkins University	1	60	57	113	3471
University of California, San Francisco	1	52	58	96	2308
University of Washington	1	58	50	90	2961
University of Sydney	2	32	41	85	1358
University of Michigan	1	49	41	81	1727
Columbia University	1	44	45	77	1462
University of North Carolina	1	40	33	75	1603
University of Pittsburgh	1	38	39	70	1676

**Table 5:** Core research topics information summary.

No.	Research Topic	Keywords (Co-Occurrence Counts)
C1	Health management	health (41); pressure (4); physical illness (2); education (13); survey (9); risk (5); outpatient service (10); mortality (2); Africa (44); diagnosis (3)
C2	Child health	health status (2); asthma (16); children (198); guideline (9); health care cost (4); child health (20); parent (17); adherence (2); chronic obstructive pulmonary disease (9); quality of life (189)
C3	Assistive technology	assistive technology (4); disability (22); cerebral palsy (2)
C4	Pharmacokinetics	muraglitazar (2); lc-ms/m (8); pharmacokinetics (28); anti-psychotic (2)
C5	Disease prevention	obesity (73); nutrition (6); diet (6); barriers (2); schoolchildren (2); body composition (14); risk factors (7); disease prevention (40); ethnicity (2); body image (3)
C6	Risk assessment	health education (2); diabetes (56); risk assessment (12); meta-analysis (2)
C7	adolescent health	sexual health (3); young people (7); adolescent (29)
C8	Telemedicine	technology (10); telemedicine (66); HTA (42); qualitative research (94); heart failure (9); patient satisfaction (8); patient reported outcome (5)
C9	Health technology assessment	quality (2); evaluation (14); older people (84); methodology (12); HIT (76); trauma (5)
C10	Digital health technology	m-health (96); mobile phone (6); smart phone (11); task shifting (3); community health workers (3); India (4); digital health (9)
C11	Well-being method	reliability (4); systematic review (2); well-being (27); methods (12)
C12	Internet	breast cancer (5); shared decision making (2); communication (17); Internet (15)
C13	Mass spectrometry	bio-marker (2); human plasma (6); mass spectrometry (4)
C14	Data collection	data collection (2); pediatric (4); focus group (8)
C15	Electronic health record	mental illness (2); evidence-based medicine (2); electronic health record (73)
C16	Physical therapy	dementia (10); physical therapy (53); rehabilitation (24)
C17	Female health	women health (46); geriatric (2); pain (4); rural (4); medicare (2)
C18	Health screening	adult (2); validity (10); screening (21)
C19	Public health	Intervention (19); public health (49); cancer (21); tuberculosis (2); care coordination (3); health disparity (6); reproductive health (3); maternal and child health (18)
C20	Health information technology	primary care (120); empowerment (2); health promotion (53); information technology (10); implementation (20)
C21	COVID-19 prevention	mobile application (11); COVID-19 (16)

## Data Availability

The Web of Science (WoS) data can be accessed through the WoS’s official website: https://www.webofscience.com/wos/alldb/basic-search (accessed on 1 March 2021).

## References

[1] Oortwijn W., Banta D., Vondeling H., Bouter L. (1999). Identification and priority setting for health technology assessment in The Netherlands: Actors and activities. Health Policy.

[2] Eldar R. (2002). Health technology: Challenge to public health. Croat. Med. J..

[3] Garrido M.V., Gerhardus A., Røttingen J.A., Busse R. (2010). Developing health technology assessment to address health care system needs. Health Policy.

[4] Sixtieth World Health Assembly WHA60.29 Health Technologies. https://www.who.int/healthsystems/WHA60_29.pdf.

[5] Inahta Hta Glossary. http://htaglossary.net/health-technology.

[6] Banta H.D. (2018). Perspective: Some conclusions from my life in health technology assessment. Int. J. Technol. Assess. Health Care.

[7] Banta D., Jonsson E. (2009). History of HTA: Introduction. Int. J. Technol. Assess. Health Care.

[8] Sadiku M.N., Akhare Y.P., Musa S.M. (2019). Emerging technologies in healthcare: A tutorial. Int. J. Adv. Sci. Res. Eng..

[9] Dunn P., Hazzard E. (2019). Technology approaches to digital health literacy. Int. J. Cardiol..

[10] Aceto G., Persico V., Pescapé A. (2020). Industry 4.0 and health: Internet of things, big data, and cloud computing for healthcare 4.0. J. Ind. Inf. Integr..

[11] Phillips S.A., Ali M., Modrich C., Oke S., Elokda A., Laddu D., Bond S. (2019). Advances in health technology use and implementation in the era of healthy living: Implications for precision medicine. Prog. Cardiovasc. Dis..

[12] Gottge S., Menzel T., Forslund H. (2020). Industry 4.0 technologies in the purchasing process. Ind. Manag. Data Syst..

[13] Javaid M., Haleem A. (2019). Industry 4.0 applications in medical field: A brief review. Curr. Med. Res. Pract..

[14] Pai R.R., Alathur S. (2021). Bibliometric analysis and methodological review of mobile health services and applications in India. Int. J. Med. Inform..

[15] Xu H., Huang S., Qiu C., Liu S., Deng J., Jiao B., Tan X., Ai L., Xiao Y., Belliato M. (2020). Monitoring and management of home-quarantined patients with COVID-19 using a WChat-based telemedicine system: Retrospective cohort study. J. Med. Internet Res..

[16] Hynes D.M., Weddle T., Smith N., Whittier E., Atkins D., Francis J. (2010). Use of health information technology to advance evidence-based care: Lessons from the VA QUERI program. J. Gen. Intern. Med..

[17] (2008). Health Information Technology in the United States: Where We Stand. https://folio.iupui.edu/bitstream/handle/10244/784/hitreport.pdf.

[18] Wild C., Langer T. (2008). Emerging health technologies: Informing and supporting Health Policy early. Health Policy.

[19] Lupton D. (2021). Young people’s use of digital health technologies in the global north: Narrative review. J. Med. Internet Res..

[20] Penno E., Gauld R. (2017). Change, connectivity, and challenge: Exploring the role of health technology in shaping health care for aging populations in Asia Pacific. Health Syst. Reform.

[21] Sweileh W.M., Al-Jabi S.W., AbuTaha A.S., Zyoud S.E.H., Anayah F., Sawalha A.F. (2017). Bibliometric analysis of worldwide scientific literature in mobile-health: 2006–2016. BMC Med. Inform. Decis. Mak..

[22] Waqas A., Teoh S.H., Lapão L.V., Messina L.A., Correia J.C. (2020). Harnessing telemedicine for the provision of health care: Bibliometric and scientometric analysis. J. Med. Internet Res..

[23] Gu D., Li T., Wang X., Yang X., Yu Z. (2019). Visualizing the intellectual structure and evolution of electronic health and telemedicine research. Int. J. Med. Inform..

[24] Yang X., Wang X., Li X., Gu D., Liang C., Li K., Zhang G., Zhong J. (2020). Exploring emerging IoT technologies in smart health research: A knowledge graph analysis. BMC Med. Inform. Decis. Mak..

[25] Sood S.K., Rawat K.S., Kumar D. (2022). A visual review of artificial intelligence and Industry 4.0 in healthcare. Comput. Electr. Eng..

[26] Gu D., Yang X., Deng S., Liang C., Wang X., Wu J., Guo J. (2020). Tracking knowledge evolution in cloud health care research: Knowledge map and common word analysis. J. Med. Internet Res..

[27] Anjum H.F., Rasid S.Z.A., Khalid H., Alam M.M., Daud S.M., Abas H., Sam S.M., Yusof M.F. (2020). Mapping research trends of blockchain technology in healthcare. IEEE Access.

[28] Liu Z., Ren L., Xiao C., Zhang K., Demian P. (2022). Virtual reality aided therapy towards health 4.0: A two-decade bibliometric analysis. Int. J. Environ. Res. Public Health.

[29] Kim A.R., Park H.Y. (2021). Theme trends and knowledge-relationship in lifestyle research: A bibliometric analysis. Int. J. Environ. Res. Public Health.

[30] Dang Q., Luo Z., Ouyang C., Wang L. (2021). First Systematic Review on Health Communication Using the CiteSpace Software in China: Exploring Its Research Hotspots and Frontiers. Int. J. Environ. Res. Public Health.

[31] Cobo M.J., López-Herrera A.G., Herrera-Viedma E., Herrera F. (2011). An approach for detecting, quantifying, and visualizing the evolution of a research field: A practical application to the Fuzzy Sets Theory field. J. Informetr..

[32] Small H. (1999). Visualizing science by citation mapping. J. Am. Soc. Inf. Sci..

[33] Small H. (1973). Co-citation in the scientific literature: A new measure of the relationship between two documents. J. Am. Soc. Inf. Sci..

[34] Callon M., Courtial J.P., Turner W.A., Bauin S. (1983). From translations to problematic networks: An introduction to co-word analysis. Soc. Sci. Inf..

[35] Mora L., Deakin M., Reid A. (2019). Combining co-citation clustering and text-based analysis to reveal the main development paths of smart cities. Technol. Forecast. Soc. Chang..

[36] Wang L., Xia E., Li H., Wang W. (2019). A bibliometric analysis of crowdsourcing in the field of public health. Int. J. Environ. Res. Public Health.

[37] Li J., Mao Y., Ouyang J., Zheng S. (2022). A Review of Urban Microclimate Research Based on CiteSpace and VOSviewer Analysis. Int. J. Environ. Res. Public Health.

[38] Eck N.J.V., Waltman L. (2010). Software survey: VOSviewer, a computer program for bibliometric mapping. Scientometrics.

[39] Chen C. (2006). CiteSpace II: Detecting and visualizing emerging trends and transient patterns in scientific literature. J. Am. Soc. Inf. Sci. Technol..

[40] Callon M., Courtial J.P., Laville F. (1991). Co-word analysis as a tool for describing the network of interactions between basic and technological research: The case of polymer chemsitry. Scientometrics.

[41] Law J., Bauin S., Courtial J., Whittaker J. (1988). Policy and the mapping of scientific change: A co-word analysis of research into environmental acidification. Scientometrics.

[42] Page M.J., McKenzie J.E., Bossuyt P.M., Boutron I., Hoffmann T.C., Mulrow C.D., Shamseer L., Tetzlaff J.M., Akl E.A., Brennan S.E. (2021). The PRISMA 2020 statement: An updated guideline for reporting systematic reviews. BMJ.

[43] Qi B., Jin S., Qian H., Zou Y. (2020). Bibliometric analysis of chronic traumatic encephalopathy research from 1999 to 2019. Int. J. Environ. Res. Public Health.

[44] Selva-Pareja L., Ramos-Pla A., Mercadé-Melé P., Espart A. (2022). Evolution of Scientific Production on Health Literacy and Health Education—A Bibliometric Analysis. Int. J. Environ. Res. Public Health.

[45] Zhao Y., Guo J., Bao C., Liang C., Jain H.K. (2020). Knowledge graph analysis of human health research related to climate change. Int. J. Environ. Res. Public Health.

[46] Price D.J. (1986). Little Science, Big Science... and Beyond.

[47] Scott B.K., Miller G.T., Fonda S.J., Yeaw R.E., Gaudaen J.C., Pavliscsak H.H., Pamplin J.C. (2020). Advanced digital health technologies for COVID-19 and future emergencies. Telemed. E-Health.

[48] Aminullah E., Erman E. (2021). Policy innovation and emergence of innovative health technology: The system dynamics modelling of early COVID-19 handling in Indonesia. Technol. Soc..

[49] Magrabi F., Ammenwerth E., Craven C.K., Cresswell K., DeKeizer N.F., Medlock S.K., Georgiou A. (2021). Managing Pandemic Responses with Health Informatics—Challenges for Assessing Digital Health Technologies. Yearb. Med. Inform..

[50] Mukherjee K. (2021). Relevance of the newly defined Health Technology Assessment: COVID-19 and beyond. Int. J. Technol. Assess. Health Care.

[51] Mahajan S., Lu Y., Spatz E.S., Nasir K., Krumholz H.M. (2021). Trends and predictors of use of digital health technology in the United States. Am. J. Med..

[52] Zaresani A., Scott A. (2020). Does digital health technology improve physicians’ job satisfaction and work–life balance? A cross-sectional national survey and regression analysis using an instrumental variable. BMJ Open.

[53] Sun X., Yan W., Zhou H., Wang Z., Zhang X., Huang S., Li L. (2020). Internet use and need for digital health technology among the elderly: A cross-sectional survey in China. BMC Public Health.

[54] Marsch L.A. (2021). Digital health data-driven approaches to understand human behavior. Neuropsychopharmacology.

[55] Dhingra D., Dabas A. (2020). Global strategy on digital health. Indian Pediatrics.

[56] Ye N., Kueh T.B., Hou L., Liu Y., Yu H. (2020). A bibliometric analysis of corporate social responsibility in sustainable development. J. Clean. Prod..

[57] Pfeiffer S. (2017). The vision of “Industrie 4.0” in the making—A case of future told, tamed, and traded. Nanoethics.

[58] Dragicevic N., Ullrich A., Tsui E., Gronau N. (2019). A conceptual model of knowledge dynamics in the industry 4.0 smart grid scenario. Knowl. Manag. Res. Pract..

[59] Mariani M., Borghi M. (2019). Industry 4.0: A bibliometric review of its managerial intellectual structure and potential evolution in the service industries. Technol. Forecast. Soc. Chang..

[60] Asif M. (2020). Are QM models aligned with Industry 4.0? A perspective on current practices. J. Clean. Prod..

[61] Bruzzone A., Massei M., Sinelshnkov K. (2020). Enabling strategic decisions for the industry of tomorrow. Procedia Manuf..

[62] Larrucea X., Moffie M., Asaf S., Santamaria I. (2020). Towards a GDPR compliant way to secure European cross border Healthcare Industry 4.0. Comput. Stand. Interfaces.

[63] Tortorella G.L., Fogliatto F.S., Mac Cawley Vergara A., Vassolo R., Sawhney R. (2020). Healthcare 4.0: Trends, challenges and research directions. Prod. Plan. Control..

[64] Jayaraman P.P., Forkan A.R.M., Morshed A., Haghighi P.D., Kang Y.B. (2020). Healthcare 4.0: A review of frontiers in digital health. Wiley Interdiscip. Rev. Data Min. Knowl. Discov..

[65] Hathaliya J.J., Tanwar S., Tyagi S., Kumar N. (2019). Securing electronics healthcare records in healthcare 4.0: A biometric-based approach. Comput. Electr. Eng..

[66] Tortorella G.L., Fogliatto F.S., Espôsto K.F., Vergara A.M.C., Vassolo R., Mendoza D.T., Narayanamurthy G. (2020). Effects of contingencies on healthcare 4.0 technologies adoption and barriers in emerging economies. Technol. Forecast. Soc. Chang..

[67] Tanwar S., Parekh K., Evans R. (2020). Blockchain-based electronic healthcare record system for healthcare 4.0 applications. J. Inf. Secur. Appl..

[68] Estrela V.V., Monteiro A.C.B., França R.P., Iano Y., Khelassi A., Razmjooy N. (2018). Health 4.0: Applications, management, technologies and review. Med. Technol. J..

[69] Dimitrov D.V. (2016). Medical internet of things and big data in healthcare. Healthc. Inform. Res..

[70] Islam S.R., Kwak D., Kabir M.H., Hossain M., Kwak K.S. (2015). The internet of things for health care: A comprehensive survey. IEEE Access.

[71] Kwon S., Kim H., Yeo W.H. (2021). Recent advances in wearable sensors and portable electronics for sleep monitoring. Iscience.

[72] Dinh-Le C., Chuang R., Chokshi S., Mann D. (2019). Wearable Health Technology and Electronic Health Record Integration: Scoping Review and Future Directions. JMIR Mhealth Uhealth.

[73] Suver C., Kuwana E. (2021). mHealth wearables and smartphone health tracking apps: A changing privacy landscape. Inf. Serv. Use.

[74] Cerchione R., Centobelli P., Riccio E., Abbate S., Oropallo E. (2022). Blockchain’s coming to hospital to digitalize healthcare services: Designing a distributed electronic health record ecosystem. Technovation.

[75] Esmaeilzadeh P. (2022). Benefits and concerns associated with blockchain-based health information exchange (HIE): A qualitative study from physicians’ perspectives. BMC Med. Inform. Decis. Mak..

[76] Kuo T.T., Kim H.E., Ohno-Machado L. (2017). Blockchain distributed ledger technologies for biomedical and health care applications. J. Am. Med. Inform. Assoc..

[77] Fatoum H., Hanna S., Halamka J.D., Sicker D.C., Spangenberg P., Hashmi S.K. (2021). Blockchain integration with digital technology and the future of health care ecosystems: Systematic review. J. Med. Internet Res..

[78] Chattu V.K. (2021). A review of artificial intelligence, big data, and blockchain technology applications in medicine and global health. Big Data Cogn. Comput..

[79] Zhuang Y., Chen Y.W., Shae Z.Y., Shyu C.R. (2020). Generalizable layered blockchain architecture for health care applications: Development, case studies, and evaluation. J. Med. Internet Res..

[80] Ichikawa D., Kashiyama M., Ueno T. (2017). Tamper-resistant mobile health using blockchain technology. JMIR Mhealth Uhealth.

[81] Abunadi I., Kumar R.L. (2021). Blockchain and business process management in health care, especially for COVID-19 cases. Secur. Commun. Netw..

[82] The Lancet (2017). Artificial intelligence in health care: Within touching distance. Lancet.

[83] Shafqat S., Kishwer S., Rasool R.U., Qadir J., Amjad T., Ahmad H.F. (2020). Big data analytics enhanced healthcare systems: A review. J. Supercomput..

[84] Jameson J.L., Longo D.L. (2015). Precision medicine-personalized, problematic, and promising. N. Engl. J. Med..

[85] Smith H. (2021). Clinical AI: Opacity, accountability, responsibility and liability. AI Soc..

[86] Ruan Q., Yang K., Wang W., Jiang L., Song J. (2020). Clinical predictors of mortality due to COVID-19 based on an analysis of data of 150 patients from Wuhan, China. Intensive Care Med..

[87] Alimadadi A., Aryal S., Manandhar I., Munroe P.B., Joe B., Cheng X. (2020). Artificial intelligence and machine learning to fight COVID-19. Physiol. Genom..

[88] Javaid M., Haleem A., Vaishya R., Bahl S., Suman R., Vaish A. (2020). Industry 4.0 technologies and their applications in fighting COVID-19 pandemic. Diabetes Metab. Syndr. Clin. Res. Rev..

[89] Bélisle-Pipon J.C., Couture V., Roy M.C., Ganache I., Goetghebeur M., Cohen I.G. (2021). What makes artificial intelligence exceptional in health technology assessment?. Front. Artif. Intell..

[90] Hendrix N., Veenstra D.L., Cheng M., Anderson N.C., Verguet S. (2022). Assessing the Economic Value of Clinical Artificial Intelligence: Challenges and Opportunities. Value Health.

[91] Rowe J.P., Lester J.C. (2020). Artificial intelligence for personalized preventive adolescent healthcare. J. Adolesc. Health.

[92] Paul S., Riffat M., Yasir A., Mahim M.N., Sharnali B.Y., Naheen I.T., Rahman A., Kulkarni A. (2021). Industry 4.0 applications for medical/healthcare services. J. Sens. Actuator Netw..

[93] Yu W., Choi J. (2020). Human identification in health care systems using mobile edge computing. Trans. Emerg. Telecommun. Technol..

[94] Lakshmi G.J., Ghonge M., Obaid A.J. (2021). Cloud based iot smart healthcare system for remote patient monitoring. EAI Endorsed Trans. Pervasive Health Technol..

[95] Alabdulatif A., Khalil I., Forkan A.R.M., Atiquzzaman M. (2018). Real-time secure health surveillance for smarter health communities. IEEE Commun. Mag..

